# Methods for Reducing the Toxicity of Metal and Metal Oxide NPs as Biomedicine

**DOI:** 10.3390/ma13020279

**Published:** 2020-01-08

**Authors:** Olga Długosz, Krzysztof Szostak, Anita Staroń, Jolanta Pulit-Prociak, Marcin Banach

**Affiliations:** Faculty of Chemical Engineering and Technology, Cracow University of Technology, 24 Warszawska St., 31-155 Cracow, Poland; OlgaDlugosz@interia.pl (O.D.); kszostak7@gmail.com (K.S.); anilos@chemia.pk.edu.pl (A.S.); jolantapulit@indy.chemia.pk.edu.pl (J.P.-P.)

**Keywords:** silver, gold, zinc oxide, titanium oxide, drug delivery, nanoparticles, medicine, reducing toxicity

## Abstract

The rapid development of medicine has forced equally rapid progress in the field of pharmaceuticals. In connection with the expensive and time-consuming process of finding new drugs, great emphasis is put on the design and use of metal and metal oxides nanoparticles in nanomedicine. The main focus is on comprehensive presentation of both physicochemical properties and the possibilities of using, in particular, silver (Ag) and gold (Au) nanoparticles, as well as zinc oxide (ZnO) and titanium oxide (TiO_2_) nanoparticles as drug carriers and in the treatment of cancer. An important element of this subject is the possibility of occurrence of toxic effects of these nanoparticles. For this reason, possible mechanisms of toxic actions are presented, as well as methods used to reduce their toxicity to ensure the safety of drug carriers based on these nanostructures.

## 1. Introduction

Over the last decade, nanotechnology has found particular interest among scientists as well as industry representatives. This field has become one of the most dynamically developing branches of science. The main reason for such intensive development is that the use of nanotechnology enables obtaining materials based on particles whose size does not exceed 100 nm in each dimension, which translates into the possibility of obtaining products with specific properties. Such materials include, for example, silver and gold nanoparticles, as well as zinc oxide and titanium oxide nanoparticles, which are the main subject of this work. Nanometric particle sizes are comparable to naturally occurring molecules and biomolecules, while being significantly smaller than human cells (~7 µm) [1]. Reducing the particle size to such a level increases the surface area of the material, making the active surface much larger which directly translates into the improvement of the chemical, electrical, magnetic, structural, or morphological properties of the substance. For this reason, nanomaterials often gain completely different properties in relation to their macro counterparts, while finding applications in many areas both in everyday life as well as in those which are more high-tech [2,3].

The very fast development of civilization forces equally rapid progress in the medical field, which also influences the use of nanotechnology for medical application. The drug market occupies an important position in the medical industry [4,5]. There is still a lot of emphasis on the development of new drugs, but from a technological point of view, the route that new active substances must follow from development to launch is very expensive and time-consuming. That is why the use of known and tested active substances on new carriers or in a new form is more preferable, because the development of new drug delivery tools is a faster and cheaper method that will allow the drug to be delivered to the right place in the body more accurately and safely [6].

**Figure 1:**
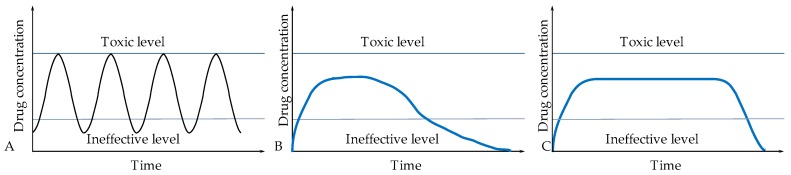
Possibilities of active substance release: (**A**) immediate release of active substance, (**B**) prolonged release of active substance, and (**C**) controlled release of active substance.

There are many ways to give medicines to patients. They depend on the disease being treated, the effect expected and the type of medicinal product. One of the oldest and most commonly used methods of drug administration is oral [7]. Its popularity is due to the ease of administration and dosage, and at the same time it is preferred by patients due to the high comfort of intake. Intravenous is another widely used method and many drugs are designed specifically for this form of administration [8,9]. However, as well as with oral administration, the active substance must travel a long way to the target site, and thus can be deactivated by the intermediate organs (stomach, liver, or kidneys) resulting in poor activity at the target organ [10]. The second quite important issue of such forms of drug administration is the relatively high initial concentration of active substances administered, which may even exceed the toxicity limit. Then this concentration drops at a fairly rapid pace, reaching a level below therapeutic value, making the treatment ineffective (Figure 1). It is for the above-mentioned reasons that it is so important to constantly search for new forms of drug administration that will allow more effective, selective, and targeted action of active substances [11]. 

Drug carriers based on metal and metal oxide nanoparticles are an alternative to currently used inorganic drug carriers [12,13]. The advantage of their use is the ability to modify their surface by creating bonds between the active substance and the carrier which allows the gradual release of the active substance after entering the body [14]. An additional advantage of using metal and metal oxide nanoparticles is the ability to simultaneously use their properties to obtain a multifunctional material, which limits the amount of used drugs [15]. On the other side, it is necessary to determine the possible undesirable effects of the interaction between nanoparticles and organisms. Due to the limited knowledge about them, such as the long-term effects of nanoparticles on organisms and the environment, including the possibility of their accumulation, it is necessary to limit the possible negative effects. An example of protecting the body against the negative effects of nanoparticles is the use of a protective coating against the uncontrolled release of metal ions into the cells and tissues. Ungor et al. compared the role of thiol and amine groups on the optical properties of gold nanoparticles. Functional groups act both as stabilizers and reducers in synthesis of gold nanoparticles. The products obtained in this way were characterized by high stability and, what is significant, high biocompatibility [16].

This paper describes the methods of production and action of drug carriers based on silver and gold nanoparticles, as well as zinc and titanium oxides. At the same time, when using nanoparticles as carriers, the possibility of toxic effects on the body should be taken into account, which is why the focus is also on methods of reducing their toxicity.

## 2. Oxide Nanoparticles as Drug Carriers

Depending on the structure, metal oxides may exhibit metallic, semiconductor, or insulating properties. In nanoparticle form, due to their small size, their energy structure is characterized by band gap values that ensure the semiconductor properties of these materials. When irradiating semiconductor materials with UV light, the electron (e-) absorbing energy is excited into the conductivity band, thereby creating an electron hole (h+) in the valence band. Thanks to this, these materials can have very good photocatalytic, photoluminescent, antibacterial, and antifungal properties, not found in their macro forms [17]. A significant number of researchers have shown that nanomaterials, including metal oxide nanoparticles, can be lethal to cancer cells, unlike their macro counterparts, which remain neutral to cancerous changes. It has also been proven with in vitro studies that specific oxide nanoparticles, in this case zinc oxide (ZnO), can act selectively on cancer cells, with significantly lower toxicity to normal cells. Due to these specific properties, they show promising perspectives and are becoming a more and more frequent research target in biomedical applications as carriers for proteins, peptides, targeted cancer drugs, biosensors, or in diagnostics as contrast agents [1]. 

Examples of nanomaterials used in diagnostic tests include iron nano oxide, which in its specific form has superparamagnetic properties, and is why it is used in imaging techniques such as magnetic resonance imaging [18]. Due to its unique magnetic properties, it can be used in theranostics, which involves the simultaneous detection of disease processes in the patient’s body and taking action that allows the delivery of drugs directly to areas covered by the disease process [19]. 

The most common oxide nanoparticles used as drug carriers are ZnO and titanium oxide (TiO_2_). They are considered as “GRAS” (generally recognized as safe) by the US Food and Drug Administration and by the International Agency for Research on Cancer. However, this applies to macromolecular materials [20]. It should be noted that due to their small size, the nanoparticles have a much larger surface area to volume ratio, and thus highly increased activity. They may also have completely different properties in relation to their macro counterparts, including unfavorable ones [21].

### 2.1. ZnO Nanoparticles

Zinc oxide in nanoparticulate form is an n-type semiconductor characterized by a band gap of 3.37 eV and excitation energy at room temperature of 60 meV [22]. Due to these parameters, nano-ZnO perfectly absorbs light in the UV range, which directly translates into excellent photocatalytic properties [23]. In addition, it has unique electrical and optical properties. These properties can be easily adapted to specific needs by controlling synthesis parameters, and thus achieving the right size and shape of particles or modifications by doping with other materials. Thanks to such solutions, it is widely used in paints, photocatalysis, piezoelectric devices, various types of sensors (UV, O_3_, and glucose), and even in solar cells. It also absorbs ultraviolet in the B range (280–315) and in the A range (315–400 nm) radiation bands harmful to humans, thanks to which it is very widely used in cosmetics and as a UV radiation blocker in tanning creams [24]. 

Due to their photocatalytic properties ZnO nanoparticles (NPs) have the ability to generate reactive oxygen species (ROS) relatively easily, which translate into good antibacterial properties. ZnO NPs have a positive surface charge and consequently they can easily combine with negatively charged bacterial membranes, which facilitates penetration into the cells, allowing them to be eradicated. These features enabled the use of these materials as excellent additives for bactericides used in dentistry and everyday cosmetics products. ZnO NPs have an isoelectric point at pH ~9. At pH values <9, ZnO NPs are surface-protonated to the form of ZnOH^2+^, which means that they remain positively charged in the pH range corresponding to body fluids, and helps in their use as drug carriers [23]. Combined photoluminescent features, used in biosensing, with antibacterial properties, promising parameters are obtained in terms of their use in theranostics. More than 10 years ago, Zhang et al. presented the use of ZnO in self-lighting photodynamic therapy (SLPDT) against human ovarian cancer (OVCAR-3) cells in culture. They proved that in dark conditions, the ZnO NPs conjugated with the methylthioadenosine phosphorylase (MTAP) anti-cancer drug to safely reach its intended site, and when exposed to ultraviolet A (UVA) radiation they were able to produce ROS, which significantly reduced the viability of cancer cells. Cells died both by apoptosis and necrosis [25]. Those studies produced clear evidence that the use of ZnO NPs in combination with already known anti-cancer drugs could deliver promising effects in cancer therapy.

### 2.2. TiO_2_ Nanoparticles

Titanium oxide, known as titania, occurs naturally in three crystal structures: Rutile, anatase, and brucite. Among them, the most stable and most commonly used form is rutile, however, looking at nanoparticles, the most preferred form is anatase. TiO_2_ NPs are characterized by a refractive index both in the UV and visible band which is why they are widely used in optical techniques. TiO_2_, like ZnO, is an n-type semiconductor material with an energy gap of 3.03 eV and 3.2 eV for rutile and anatase, respectively [19]. At a relatively low cost, it is highly chemically stable, non-toxic, biocompatible, and has high activity. It has similar properties to ZnO. It is the most commonly used material in photocatalytic applications. It is also used in a wide range of different products, among others, in the paint and food industry, as well as in more specialized branches such as dye-sensitized solar cells, and gas sensor devices [26]. The above-mentioned properties translate into the possibility of using TiO_2_ NPs in drug carriers, improving their effectiveness. As with most nanomaterials, preparation techniques are the main factors affecting the final properties of the material. Depending on the parameters and the method used, it is possible to control the shape and size of the particles. A number of methods are currently known for the preparation of TiO_2_ NPs. Among them, the most popular are sol-gel, hydro, and solvothermal methods as well as electrochemical methods [11]. Zhang et al. investigated the possibility of using TiO_2_ NPs as a drug carrier for daunorubicin (DNR), which responds to changes in pH. Despite the fact that DNR is a powerful anti-cancer drug, its use is limited due to its strong side effects. That is why their goal was to load DNR into TiO_2_ NPs for the controlled release of DNR. They examined the degree of drug release, the process of ingestion by cells and the cytotoxicity of the obtained nanocomposite. It was noted that lowering the pH from 7.4 to 5.0 accelerated the release of DNR. At the same time, it was found that the absorption of the drug into the cells was increased from the nanocomposite obtained compared to the pure active substance. MTT (3-(4,5-dimethylthiazol-2-yl)-2,5-diphenyltetrazolium bromide) cytotoxicity tests on leukemia cells (K562) showed that pure TiO_2_ NPs were non-toxic. The active substance, DNR, showed toxic effects as expected, however, when combined with TiO_2_ this effect was even stronger. Researchers explained that the main impact on this is the pH of the environment, because at pH 7.4 the active substance is still bound to nanoparticles, but as the pH decreases, protonation of TiO_2_ NPs occurs, which directly translates into the release of the drug from conjugates [27]. Due to this mechanism, it is possible to release a precise large dose of DNR straight into the cancer cells, leading to their death [28]. These studies have shown that it is possible to improve the site of action of active substances in cancer drugs by controlling the pH of the environment.

### 2.3. Other Oxide Nanoparticles

Other types of oxide nanoparticles that are gaining interest in biomedical applications and among drug carriers include the above-mentioned iron oxide nanoparticles with good magnetic properties. Among iron oxide nanoparticles, superparamagnetic iron oxide nanoparticles (SPIONS) consisting of γ-Fe_2_O_3_ and Fe_3_O_4_ stands out. They are mainly used as contrast in imaging (MRI), but are also found in targeted drug carriers and biosensors. SPIONS toxicity studies show that they may be mutagenic and neurotoxic, induce genotoxicity, and also cause adverse effects when subject to long-term exposure [19].

Other nanoparticles of interest in biomedical applications also include cerium and silica nanoparticles. The former are particularly interesting because of their unique ability to change the degree of oxidation, so they become a good catalyst and antioxidant. When moving to silica nanoparticles, their highly porous structure, developed surface, and good biocompatibility, as well as relatively easy control of particle size, should be taken into account. Both of these materials have good predispositions as drug carriers, biosensors, and even anti-cancer agents [28]. 

## 3. Metal Nanoparticles as Drug Carriers

One of the last solutions offering hope in chemotherapy is the use of metal nanoparticles as drug carriers [29]. One of the basic advantages of choosing metal nanoparticles as drug carriers is their ability to increase the solubility of hydrophobic medicinal compounds, increasing the dissolution efficiency of the active substance in the blood. An additional advantage is the enhancement of the action of medicinal substances against cancer cells, thus reducing the need to administer higher concentrations of dangerous drugs [30]. The use of nanoparticles obtained by biological methods also increases the biocompatibility of particles. In addition, due to the small size of the nanoparticles, they help active substances pass through biological barriers and release the drug in the right place [31].

Despite the many advantages, the use of combined nanometal-drug particles is difficult. The main disadvantages of these solutions include increasing the cost of therapy due to the use of metallic nanoparticles, complicating the process of obtaining the final drug through the multi-stage process and the possible toxic effects of the nanometals used [32]. An important challenge facing nanometallic media is their ability to bring active compounds to the required place with sufficient efficiency. Due to significant forces occurring between the particles and the medium, their agglomeration may occur [33]. In addition, metallic nanoparticles themselves can cause harmful effects, especially on healthy cells.

Drug immobilization on nanocarriers is carried out using physical processes (adsorption and encapsulation) or chemical methods (using covalent, ionic, van der Waals bonds between the drug and the carrier). Due to the possibility of modifying metal surfaces with appropriate functional groups, it is possible to create suitably strong carrier-drug connections. The presence of additional compounds on the one hand gives nanoparticles a multifunctional character, which is a key element in modern targeted therapy, on the other it extends the drug delivery process [34], ensuring even drug release. Due to their optical, magnetic, and thermal properties, nanometals can be used in drug delivery, medical diagnostics, autotherapy, and many others [35]. Combining them with the drug creates a synergistic effect of diagnosis and therapy, which minimizes the need to administer various chemical compounds with single functions. The possibility of imaging with the simultaneous use of active substances allows real-time therapy to be monitored, allowing rapid response in the event of disease changes.

The unique properties of nanometals are associated with their small size, and thus a high ratio of surface to volume of particles. In addition, depending on the method by which the metal nanoparticles are obtained, it is possible to obtain particles that differ not only in size but also in shape, which also affects their properties [36]. Gold and silver nanoparticles are by far the most important in medical applications. Due to their low toxicity, ease of preparation, the ability to modify their surface, and good stability, they offer the greatest hope for use as drug carriers. In recent years, nanoparticles of bismuth, gadolinium, or selenium, which are characterized by high biocompatibility and non-toxicity, are also gaining more and more importance [37]. Due to the high requirements for medicines, before their introduction into widespread use, it is necessary to conduct research to confirm the possibility of their use without adverse effects on organisms.

The most common metallic nanoparticles used as drug carriers are gold and silver nanoparticles. In addition to high stability and the possibility of obtaining nanoparticles with a narrow size distribution and variable morphology, these nanoparticles have a number of additional properties, including optical, thermal, antimicrobial or catalytic, which have become the subject of intensive research, especially of using these particles in the field of oncology and therapy [38]. It has also been proven that nanoparticles alone can enhance the effects of medicinal substances and act as active compounds themselves. It is important that nanoparticles remain stable throughout treatment and that nanoparticle size does not exceed 100 nm to avoid opsonization and subsequent elimination from the immune system [39]. Both gold and silver nanoparticles may also have some disadvantages, in particular the possibility of releasing metal ions into the environment, which affects their cytotoxicity. Despite the concerns related to the use of metal nanoparticles, it is possible to provide a high-quality, non-toxic product by using a number of methods that modify their structure [40].

### 3.1. Silver Nanoparticles

Silver nanoparticles (AgNPs) have very good antibacterial, antifungal, and antiviral properties. Due to the easy synthesis, many methods of obtaining AgNPs have been developed, including the increasingly used biological methods, thereby obtaining a number of products that differ in size, shape, stability, and thus physical and chemical properties [29]. Due to the ease of obtaining small-size nanoparticles and well-defined shapes, AgNPs have been used as drug carriers in drug delivery systems. By choosing the right methods of synthesis, reagents, and stabilizers used, it is possible to obtain a product that facilitates the transport of active substances and allows the gradual release of drugs into the cells [41]. AgNPs also have some disadvantages, i.e., they can be toxic to both diseased and healthy cells. Boudreau et al. evaluated partial and ionic forms of silver and particle size for differences in silver accumulation, distribution, morphology, and toxicity. Differences in the structure, distribution, and morphology of nanoparticles and silver ions were observed. Ag NPs appeared mainly in cells, while silver ions showed affinity for extracellular membranes. The authors observed the effect of both Ag NP size and silver concentration on the degree of silver accumulation in tissues and organs, with silver accumulation occurring mainly in the kidneys, liver, jejunum, and colon [42]. Due to the activity of Ag NPs and ions and their accumulation, silver-based carriers can pose a great threat to healthy cells [43]. It is therefore necessary to minimize potential toxic properties while retaining the benefits of using silver-based carriers.

In addition to strong antimicrobial properties, Ag NPs show anti-cancer activity. Their high antibacterial, antifungal, and antiviral activity results from the activity of the nanoparticles themselves, the release of silver ions and the generation of reactive oxygen species. This may cause restrictions on the use of pure Ag NPs. To solve the problems associated with this, polymer coatings are used, which are an effective way to reduce the apparent toxicity of nanoparticles. Petrow et al. obtained core-shell nanoparticles obtained by coating Ag NPs with a cross-linked poly (L-lysine) coating containing doxorubicin. Research results showed good biocompatibility and induction of cancer cell death after drug release [44]. In addition, the modification of Ag NPs can contribute to the gradual release of the active substance, without reducing the stability of nanoparticles residing for a long time in a reactive environment. The results of research on Lidocaine Ibuprofen (IL) stabilized silver stabilized nanoparticles in combination with Ibuprofen showed that the time to the start of action by IL-Ag NPs is 10 min, which is much longer than in the case of eutectic mixture of local anesthetics (EMLA). Test results also confirmed the stronger and faster effect of IL-AgNP local anesthesia compared to EMLA [45]. Mandal et al. developed a new biopolymeric nanocomposite hydrogel composed of formed in situ silver nanowires, deposited on carboxymethyl cellulose (CMC). The biopolymeric carrier showed better efficacy as a curcumin anti-cancer carrier. The advantage of the biopolymer nanocarrier was the ability to encapsulate both hydrophobic and hydrophilic transdermal drugs. An ex vivo study of rat skin penetration confirmed that the drug was permeable in a controlled manner [46]. The possibility of producing such nanocomposites as polystyrene-silver, obtained by biological methods, has also been proven in terms of their use as carriers of non-toxic drugs. Jabbar et al. described methods using a number of plant extracts to obtain silver-based nanocarriers. The use of one-step methods in combination with biocompatible compounds contained in green leaves resulted in a stable product with high efficiency in drug delivery, without a cytotoxic effect on healthy cells [43]. In the case of polymer coatings, there is a risk of particles not closing up, especially those with low molecular weight due to their porous and network structure. By connecting AgNO_3_ salts to the polymer coating additional surfactants and L-ascorbic acid Sun et al. received a low cytotoxic carrier for up to 10 capsules per cell without releasing AgNPs [47].

Using the optical properties of Ag NPs, it is possible to obtain multifunctional carriers. By combining the benefits of optical imaging and surface-enhanced Raman scattering (SERS) detection with hyperthermia due to site specificity, Ag NP-based drugs can be an alternative to targeted medicine. Boca-Farcau et al. used silver nanotriangles that were biocompatible with chitosan biopolymer, with embedded para-aminothiophenol (pATP) molecules and conjugated with folic acid. In studies on ovarian cancer cells NIH: OVCAR-3, conjugated particles have proven to be highly stable in aqueous and cellular environments. Targeted uptake of conjugated nanoparticles by human ovarian cancer cells showed an effective therapeutic response compared to similar pure nanoparticles [39].

One of the benefits of using AgNPs is their small size. In most cases, particles smaller than 100 nm in diameter are suitable for drug delivery applications. After coating nanoparticles with active substances, the size of the entire composite increases significantly, hence it is so important that the carrier itself is small. The smaller the Ag particle is, the greater the accumulation in malignant places, which is also associated with better transport across different types of cell membranes. On the other hand, smaller particles can also increase intracellular cytotoxicity with their degradation. Because Ag particles are neutral, their cytotoxicity is less than that of charged particles. The attraction force between the conducting electrons of Ag particles and the membrane of malignant cells promotes the rate of adhesion to the target surface of the diseased cell, initiating the toxic effect. Small Ag particles, with a diameter smaller than 50 nm, effectively change the potential of malignant cells and also impede their proliferation [34]. The small size of Ag NPs can help fight the most dangerous cancer cells. Liang et al. investigated the effect of Ag NPs on human glioblastoma U251 cells and their role as a carrier of temozolomide (TMZ), an imidazotetrazine derivative of the dacarbazine alkylating agent. Particles with an average size of 26 nm showed dose-dependent cytotoxicity on U251 cells. They also showed TMZ ability to increase drug sensitivity in U251 cells, which confirmed the possible synergistic effect of Ag NPs—an active substance in enhancing glioma chemotherapy [48].

### 3.2. Gold Nanoparticles

Gold nanoparticles (Au NPs) are another type of nanoparticle used in biomedicine. In the literature they are described as convenient drug delivery nanosystems also used in hyperthermia and biosensors [49]. Of the inorganic nanoparticles, Au NPs are most often studied due to their unique optical-electronic properties, ease of synthesis, surface modification, non-toxicity, and biocompatibility [50]. To minimize the potential effect of cytotoxicity and increase their biocompatibility, a number of natural compounds such as rubbers, proteins, glucan, and biopolymers, e.g., chitosan, are used [38]. An additional advantage is the ability to combine the functions of drug carriers with simultaneous diagnostic imaging. Coating nanoparticles with biopolymers, including heparin, chitosan, dextran, or cellulose does not block the activity of nanoparticles, but only improves their biocompatibility and stability, so that the material remains multi-functional. Sahoo et al. developed a method for obtaining high fluorescence Au NPs using chitosan. Without the need for additional compounds, a product with high stability was developed in both the water and powder phase. The use of chitosan helped to easily create composite nanoparticles which was useful for delivering drugs to cancer cells in combination with easy imaging [51]. However, the biopolymers used have some disadvantages, such as low solubility and high viscosity. Laksee et al. used pullulan as a nonionic polysaccharide that showed high water solubility, non-toxicity, and biocompatibility while maintaining a low price [52]. Therefore, the main challenge is to limit the toxic effects of pure Au NPs as they have high surface binding energy with the surrounding environment, which can cause protein adsorption. To date, almost 120 human plasma proteins have been shown to bind to Au NPs. Although protein adsorption prevents rapid aggregation, it does not exclude nanoparticle-cell interactions. Consequently, the Au NP toxicity can be drastically increased. 

As with Ag NPs, Au NPs have a strong affinity for thiol-gold and amine-gold interactions [53]. By choosing the right compounds for synthesis as well as auxiliary substances or the active substances themselves, the toxicity of Au NPs can be minimized. Selective toxicity of AuNPs has been demonstrated by Prakash et al. who used the marine bacteria *Paracoccus haeundaensis* for extracellular synthesis of AuNPs and confirmed that the synthesized nanoparticles did not inhibit the growth of normal HaCaT and Human embryonic kidney HEK293 cells, while demonstrating concentration-dependent inhibition of growth in adenocarcinomic human alveolar basal epithelial cells (A549) and adenocarcinoma gastric (AGS) cancer cells [54]. The use of methoxypolyethylene glycol-graft-poly (L-lysine) copolymer (MPEG-gPLL) in combination with free amino groups, enabled a product with very low toxicity in human endothelial cells, but showed high dose-dependent toxicity in epithelial cancer cells [53].

Regardless of the scope of application of Au NPs, it is very important to maintain their stability by preventing their aggregation. Compared with other amino acids tested, phosphotyrosine significantly stabilizes Au NPs. The results indicate that the modified Au NPs showed a high level of stability in various solutions and had good biocompatibility. When pY peptide was used to stabilize Au NPs, the phosphate group could be removed with phosphatase, which then aggregated and released the Au NP charge. A significant increase in phosphatase has been observed in many types of cancer, indicating that aggregation of nanoparticles can be regulated by phosphatase [55]. It was also noted that by modifying the gold surface with polyethylene glycol modified with thiol groups, high efficiency was confirmed in the protection of Au NPs, in particular against the release of gold while allowing the binding of a large amount of drug and testing them as a carrier [56]. Masse et al. described three types of ultra-stable Au NPs stabilized by thiolated polyethylene glycol groups. Because blood is a strongly ionic medium, it can significantly reduce the quality of nanosystems. Depending on the contact time, aggregation of metal nanoparticles can take place within a few minutes of exposure to the salts contained in the medium. After several tests, such as freeze drying, heating, ultracentrifugation, and autoclaving, the high stability of nanoparticles was confirmed. In addition, ultra-stable nanoparticles did not show any signs of cytotoxicity [57].

### 3.3. Other Metal Nanoparticles

A different approach, compared to the use of single types of metallic nanoparticles, is to obtain core-shell nanoparticles that allow the acquisition of new bioactive compounds consisting of two metals. Woźniak-Budych et al. developed a method for obtaining nanoparticles based on copper, which was the core of the particles, and gold as a coating. The main advantage of the material was the high stability of the particles, especially at a pH below seven, which in the case of contact with cancer cells with a pH of usually around five to six, is a particularly advantageous feature. In addition, the porous nature of the copper-gold particle structure has facilitated the adsorption of doxorubicin. The authors showed that copper-gold nanostructures loaded with doxorubicin inhibited the proliferation and viability of cancer cells in a concentration-dependent manner. In addition, no effect of copper-Au NPs on mitochondrial function or cell mortality in human fibroblasts was observed. The observed decrease in the toxicity of copper nanoparticles and gold may be associated with a porous layer of gold on the surface of copper nanoparticles which is essentially non-toxic [58]. Mittal et al. described the method of obtaining monodisperse bimetallic nanoparticles (Ag-Se) as quercetin carriers. Various reaction parameters (concentration of quercetin, gallic acid and Ag-Se salts, pH, temperature, and reaction time) have been optimized to control the properties of nanoparticles. Synthesized Ag-Se nanoparticles were used as anti-cancer agents for Dalton lymphoma (DL) cells, and in vitro 80% of their viability was reduced at 50 µg/mL [59].

In addition to the use of silver and gold nanoparticles, a growing trend has been observed in the use of, among others, bismuth [60], selenium [61], and gadolinium nanoparticles [62]. These materials showed not only a lack of cytotoxicity, but also acted synergistically in combination with medicinal substances, which further created opportunities for their use in the treatment of cancer. For example, selenium nanoparticles (Se NPs) work well as carriers of doxorubicin (DOX) due to their good antioxidant activity and lack of toxicity due to the natural presence of selenium in the human body [31]. Examples of effective carriers are galactose modified selenium nanoparticles (GA-SeNPs) loaded with DOX and used in the treatment of hepatocellular carcinoma (HCC). GA-Se NPs successfully adsorbed doxorubicin, thus effectively acting against Human Hepatoma HepG2 cells showing significant activity in inducing HepG2 cell apoptosis in in vitro studies [37]. Yang et al. obtained carriers based on bismuth nanoparticles modified with 1,2-dilauroyl-sn-glycero-3-phosphocholine (DLPC modified). Particles with a diameter of 47 ± 3 nm have proven themselves, among others, as a teranostatic agent in phototherapy (PA), in x-ray computed tomography (CT) or in controlled photothermal therapy (PTT). Bismuth nanoparticles in combination with DLPC have been shown to be successfully accumulated in the tumor area due to the effect of increased permeability and retention. Thanks to PTT, the growth of cancer cells (MDA-MB-231 cells) can be significantly shortened in vitro and in vivo; meanwhile, no obvious damage or noticeable toxicity to major organs was detected [40].

## 4. Toxicity of Nano Drug Carriers 

The use of drug carriers based on metal and metal oxide nanoparticles, despite their great potential, has some negative effects. Problems resulting from the use of nanoparticles are associated with their reduced stability, tendency to agglomerate, the possibility of releasing metal ions, or changing the composition by oxidizing their surface. In the case of nanoparticles, the level of cytotoxicity may depend on the type of nanoparticles, i.e., their morphology (shape, size), chemical purity and type of solvent used, which are associated with the choice of the preparation method, type of functionalization, and type of biofunctionalization of nanocarriers as well as their stability and aggregation susceptibility and agglomeration [63,64]. Changes in the above parameters affect their toxicity, resulting among other things, from the way metal ions are released or from affecting healthy cells, contributing to their damage and death.

The increase in the cytotoxicity of nanoparticles depends on many factors, including route of administration and place of cumulation. Modified nanoparticles, properly synthesized, are capable of prolonged release of the drug, without adversely affecting healthy cells. The biological compatibility of nanoparticles depends on their size, structure, and composition. The main groups of factors affecting the biocompatibility of nanoparticles and their non-toxicity are as follows:

Area of application of metal nanoparticles as carriers; the metallic nanocarriers used in a particular application may be toxic to specific types of cells. However, they may not have the same effect on other types of tissue.

The half-life of nanoparticles, i.e., depending on the time of drug delivery as well as the distance travelled, these carriers may have cytotoxic properties.

Biocompatibility of drug-carrier particles, which affects their stability in the biological medium.

The environment in which nanoparticles are to be dissolved. A particularly important factor in this case is the pH of the environment, which at reduced values may cause complete dissolution of nanoparticles, preventing their operation, and causing the migration of metallic compounds throughout the body.

The mechanisms of cytotoxic activity of nanoparticles are not fully known, however, it is believed that the ROS produced are the most significant contributors. Cell defense mechanisms deal with small amounts of ROS, however, if the amount of ROS exceeds the antioxidant capacity of cells, then biomolecules are destroyed in the processes of strong oxidation, which can become a direct cause of cell death. The effect of ROS on cells has been described as a three-stage model. The first stage is characterized by increased activity of antioxidant enzymes to defend the cell. The second is the increase in the amount of proinflammatory cytokines, which leads to inflammation, and in the third, mitochondrial perturbation appears, which leads to cell death. This was described based on the observation of the toxic effects of ZnO NPs on phagocytic or bronchial epithelial cells, which was manifested in the secretion of lactate dehydrogenase, damage to DNA, proteins and lipids, and finally ended in death by apoptosis or necrosis [1].

One of the main reasons metallic nanoparticles can be toxic is the release of their ions into the environment and disbursing throughout the body. The antimicrobial activity of metal nanoparticles is directly proportional to the number of biologically active ions released and their availability for interaction with the bacterial cell wall. For example, AgNPs in the presence of sulfides and chlorides react by destroying their original structure and changing their original properties [65]. Levard et al. found that even a very low level of AgNP sulfidation can cause the formation of insoluble silver sulfides. This reduced the toxicity of silver in this form, but changed its form, which in the case of carriers may be unfavorable [66]. Metal ions, after entering the bloodstream, can diffuse into the cells, including healthy ones. The possibility of the release of metal ions from nanoparticles is due to many factors, including particle size, shape, presence of additional surface coatings, or the environment in which they are found. Free ions affect cell viability, induce oxidative stress, and cause the release of cytokines [65]. The chemical composition of the nanometallic carrier surface allows formation of a biocompatible coating for human body cells. On the one hand, the coatings protect the metallic cores of the particles against oxidation, and on the other, they allow the attachment of specific functional molecules to the core, i.e., drugs, targeting molecules, and contrast agents. Also, in the case of metal oxide nanoparticles, the mechanism of the toxic action of oxide nanoparticles is closely related to the release of individual metal ions by dissociation during contact with cells or biological fluids. The way nanoparticles get inside the cell is also important. Nanoparticles that get into the cell through endocytosis can cause much more damage to the cell compared to nanoparticles that went through a different route. It is associated with the acidic environment prevailing inside lysosomes, which facilitates the release of ions from nanoparticles, and those in reaction with the cytoplasm directly lead to the production of oxygen radicals (superoxide radicals). As a result of the defensive action of the antioxidant enzyme called superoxide dismutase (SOD), these radicals are converted into hydrogen peroxide (H_2_O_2_), and this in turn in the presence of another enzyme, catalase (CAT), is converted to water. However, the same as in the previous case, if the amount of superoxide radicals is too high in relation to CAT capabilities, part of H_2_O_2_ does not decompose, enters the nucleus and undergoes Fenton-type reactions in which ROS are generated, causing the aforementioned damage of biomolecules and, as a consequence, cell death [19].

Aggregation is another problem that affects the stability of nanoparticles and limits their use as drug carriers. Particle collisions due to Brownian motion lead to aggregation and precipitation. Therefore, it is very desirable to obtain nanoparticles that are well dispersed and stable in the solution phase (especially in physiological serum). A possible solution is to increase the repulsion between nanoparticles, which increases their colloidal dispersion. However, in the case of nanoparticles used in biomedical therapies, chemical stability in the biological environment is hampered. For example, acidic conditions (pH = 6–7) found in cancer cells can cause aggregation of many nanoparticles. Nevertheless, methods for obtaining stable nanoparticles, also at neutral or acidic pH, are known. Shakiba et al. synthesized AuNPs with high stability under acidic conditions by creating a self-assembled monolayer to improve the interfacial properties of the particles. Increased stability results from the formation of an aqueous layer around the particles, which has contributed to increasing the dispersion of particles in the biological medium [67].

For aesthetic and marketing reasons, to achieve transparency in tanning UV protection products and creams, where both ZnO and TiO_2_ are widely used, particles smaller than 30 nm are used. Noting the very small particle size, the possibility of nanoparticles penetrating the surface layers of the skin and getting into viable cells should be considered. Fortunately, in this case, tests conducted in vitro and in vivo clearly indicate the lack of penetration of these nanoparticles into the deeper layers of the skin [68]. In addition to exposure through the skin, there are other means of getting nanoparticles into the human body, such as orally or by inhalation. Once nanoparticles enter, they can easily spread throughout the body and specific organs, as well as enter cells through phagocytic and endocytic mechanisms. Wang et al. found that ZnO nanoparticles administered orally to mice ultimately reach the liver, heart, spleen, pancreas and bone. At the same time, they noticed the decreasing toxic effect of 20 nm nanoparticles on the mouse body with an increasing dose. Hence, special attention should be paid to oral exposure to small doses of small size ZnO NPs [69].

An undoubted limitation when using metal nanoparticles and metal oxides is the lack of relevant experimental and theoretical data. Nanoparticles have gained popularity as drug carriers in a relatively short time. Currently, understanding the biological compatibility of nanoparticles requires continuous research, especially considering the effect of metallic nanoparticles on organisms after a period of prolonged use [64]. 

## 5. Methods for Reducing the Toxicity of Drug Carriers Based on Nanoparticles

**Figure 2:**
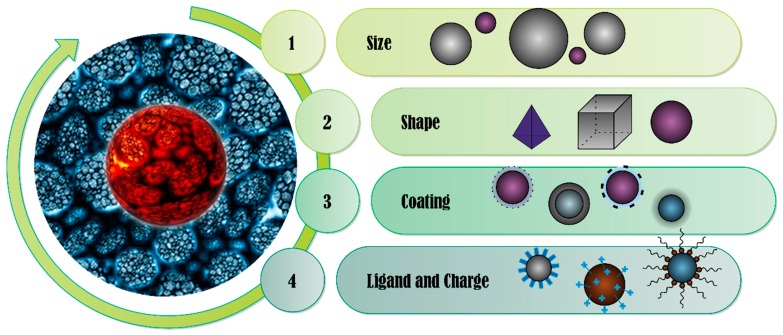
The main properties of nanoparticles affecting their toxicity.

There are many methods to prevent or limit the toxic effects of metallic nanoparticles and metal oxides. Studies have shown that changing the shape and size of particles, and methods to modify their surface, can lead to the formation of nanoparticles with the desired properties, but without a toxic effect [16]. Nanoparticles used as drug carriers are exposed to a physiological medium consisting of high levels of salt and various proteins. Both of these factors affect the stability of nanoparticles. High salt concentration reduces electrostatic repulsion between nanoparticles, leading to their aggregation, while proteins are adsorbed on the surface of nanoparticles and change the size of particles and surface charge [30]. Understanding the behavior of nanoparticles in real systems, and their possible interactions with biological systems, is crucial for the safe implementation of these materials in biomedical diagnostics and therapy. Factors that may increase the toxicity of metal nanoparticles in their application as drug carriers are shown in Figure 2.

### 5.1. Methods for the Synthesis of Metal and Metal Oxide Nanoparticles

At the stage nanoparticles are obtained it is possible to limit their potential toxic properties. The methods of producing nanoparticles allow products of various shapes and sizes to be obtained. Factors that affect these properties include process temperature, pH of the reaction, form of energy supply, choice of reagents, and reaction environment. During or after the synthesis process, substances whose task is to modify and stabilize the surface of nanoparticles are usually added to the system. 

Nanoparticles can be obtained by chemical, physical, and, increasingly, biological methods. In chemical processes, oxide nanoparticles are most often obtained in simple precipitation reactions, while nanometals are most often obtained in chemical reduction processes of metal ions using organic and inorganic reducing substances. Initially, it leads to the formation of metallic particles, which stick together to form oligomeric agglomerates. These clusters can form colloidal metallic particles. To inhibit the agglomeration process, stabilizing substances are introduced into the system or the temperature and pH of the system are controlled. In the case of metal nanoparticles intended for drug carriers, stabilizers perform two functions. First, they stabilize and protect nanoparticles against further agglomeration. Secondly, they change the nature of the surface of nanoparticles, as a result of which it is possible to adapt the surface, e.g., to ease the joining of nanoparticles with a drug. Among the physical methods, the most commonly used techniques are evaporation-condensation techniques and laser ablation in solution [70]. Despite the lack of the need for additional chemical reagents, these methods require expensive specialized equipment, hence the limited possibilities of their use.

Biological methods are becoming more and more popular. Due to the possibility of using naturally occurring reagents obtained from bacteria, fungi, algae, or plants, it is possible to obtain biocompatible products. Many methods are known in which extracts, infusions, or solutions based on natural materials have been used. Kitching et al. described methods of obtaining AuNPs using a number of species of fungi [71], and Ramkumar et al. obtained AgNPs using seaweed *Enteromorpha compressa* extract [72]. In the case of biological methods, the main limitation is obtaining homogeneous biological samples intended for the process. The use of biological methods often results in the inevitable functionalization of the surface, because the natural components used (which are reducing agents, stabilizers or other components) have an affinity for the surface of metal nanoparticles. Compounds containing sulfur, nitrogen, or oxygen easily react with metal ions initiating their reduction, and due to the close contact of compounds, the simultaneous stabilization of freshly formed nanoparticles. An example of obtaining metallic nanoparticles by the biological method is the synthesis of nano gold modified with para-aminobenzoic acid-quat-pullulan (PABA-QP) as a carrier of doxorubicin. Due to surface modification, higher drug loading was possible, which resulted in a cytotoxicity 2.1-times higher against Chago cells compared to pure doxorubicin [52]. 

### 5.2. Morphology of Metal and Metal Oxide Nanoparticles

Nanoparticles used in medicine are similar in size to cellular organelles. Depending on the size, shape, surface charge, and the presence of surface chemical groups, nanoparticles are able to penetrate the bilipid layer of the cell membrane. The cytotoxic properties of both metallic nanoparticles and metal oxides increase with decreasing particle size. This is due to the easier transport of nanoparticles across intercellular membranes. Easier access to the inside of the cell can cause the release of metal ions directly into the cell.

Depending on the choice of method, process parameters, and additional compounds used, it is possible to obtain nanoparticles with spherical, elongated, cubic, triangular, and many other shapes. This results in compounds with different surface to volume ratios. The shape of nanoparticles plays an important role both in the specification of nanoparticles for selected medicinal substances as well as in changing their toxic and immunological effects. The relationship between the influence of the shape of AgNPs on cell toxicity was demonstrated by George et al. The authors presented the results in which platelet-shaped AgNPs showed greater toxicity to fish gill epithelial cell lines (RT-W1) compared to spherical and wire-shaped nanoparticles, which had fewer surface defects [73]. Woźniak et al. determined the cytotoxicity profile of AuNPs towards normal and cancer cell lines. The authors examined AuNPs of various morphology in terms of both shape and size of particles, including nanospheres (d ≈ 10 nm), nanowires (d ≈ 41 nm), nanostars (d ≈ 240 nm), nanoflowers (d ≈ 370 nm), and nanoprisms (d ≈ 160 nm). The concentrations of the analyzed nanoparticles were tested in the range of 1–300 μM. The results showed a low cytotoxicity profile of spherical nanoparticles, especially at lower concentrations [74]. Hua et al. compared the shape effect of ZnO NPs on their toxicity and showed that nanowire-shaped particles had higher toxicity compared to spherical and cubic particles [63].

Due to the small size of nanoparticles, higher concentrations increase their cytotoxic properties. The relationship results from the increased aggregation of nanoparticles in comparison to larger-sized AuNPs (nanoparticles, nanoflowers, and nanoprisms) [73]. Sruthi and Mohanan confirmed that increasing the concentration of ZnO NPs reduces lysosomal and mitochondrial activity of glial cells, and the share of apoptosis depends on both the concentration and the time of dosing of nanoparticles [75].

### 5.3. Protective Coatings

A different approach to influencing the properties of metallic nanoparticles, including limiting their toxic effects, is to use appropriate surface modifications. By modifying the surface of metallic nanoparticles, it is possible to improve their properties or give them completely new ones, as well as stabilizing the entire carrier–drug system [73]. The main task of using coating compounds is to improve the stability of nanoparticles by preventing the release of ions from inside, preventing oxidation of the surface of nanoparticles and inhibiting aggregation, and subsequent agglomeration of nanoparticles. Sotitiou et al. found that coating AgNPs with a thin layer of SiO_2_ minimized their toxicity by blocking the release of ions and contact of bacteria and/or cells [65].

**Figure 3:**
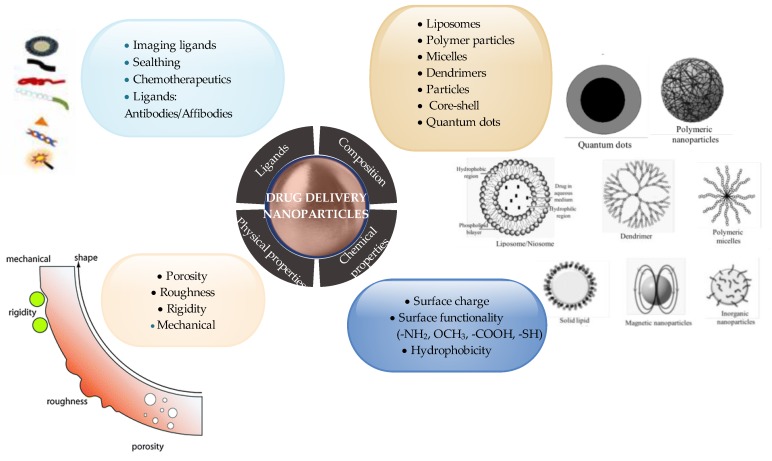
Drug delivery nanoparticles, according to the physicochemical properties and composition of nanoparticles.

Natural compounds such as saccharides, hydrocolloids, and polyphenols contained in plant extracts can be effectively used as factors improving the biocompatibility of metal nanoparticles (Figure 3). Pooja et al. described the method of obtaining AuNPs stabilized with karaya gum. The modified nanoparticles were used as the carrier of the anti-cancer drug gemcitabine hydrochloride. Rubber stabilized nanoparticles have been found to be biocompatible during cytotoxic studies and hemolysis. Stability test results suggest that the presence of rubber particles on the surface of nanoparticles not only acts as a reducing agent, but also gives nanoparticles colloidal stability [38]. Another widely used compound with high biocompatibility is chitosan, a biodegradable cationic polymer that has been successfully used in the preparation of metal nanoparticles [76]. Nayak et al. developed a method of obtaining AgNPs from *Hibiscus rosasinensis* extract in a chitosan matrix. Nanoparticles have been successfully used to encapsulate antioxidants on the examples of vitamins E and catechol acting as anticancer substances [77]. Also, for AuNPs, the use of chitosan reduced their toxicity to healthy cells. Laksee et al. examined chitosan-modified nanoparticles loaded with doxorubicin against healthy lung cells, with cell survival above 80%. Due to surface-modified Au NPs, doxorubicin was released in cancer cells. Au NPs protected the drug outside the cancer cells without causing the drug to enter healthy cells [52]. Woźniak et al. examined the cytotoxicity of Au NPs in terms of the coating agent used. The use of cetyltrimethylammonium bromide (CTAB), which is an excellent reducing agent, increases the toxic properties of the obtained nanoparticles. After the addition of L-ascorbic acid in combination with centrifugation, the Au NPs prepared in this way were not cytotoxic to selected healthy cells [74]. Mohammadzadeh confirmed the possibility of also using polymeric coatings protecting nanoparticles, in this case specifically silver. Polymer micelles are sensitive to environmental changes so that nanoparticles can be released in a controlled manner ensuring high anti-cancer effectiveness, with limited absorption into healthy tissues [78]. 

### 5.4. Surface Functionalization

In addition to coating nanoparticles with stabilizing substances whose task is to protect them, an equally important method is to functionalize the surface of nanoparticles by introducing appropriate functional groups [77]. Depending on the properties of the nanoparticles, their future use or the drug to be combined with the nanocarrier, a variety of ligands are used. In the case of drug delivery systems, such surface modifications allow the creation of appropriate mechanisms for loading and releasing the drug into target cells, changing their character to hydrophilic/hydrophobic, which improves drug solubility in the system and improves penetration through well-defined membranes. The most important groups that can be used as surface modifiers of metal nanoparticles are disulfide, amine, thiolate and dithioline, carboxylate, and phosphine groups. Luo et al., by modifying the surface of ZnO NPs with polyethylene glycol, reduced the cytotoxicity of nanoparticles while increasing their cell compatibility. The use of polyethylene glycol reduced the formation of protein crowns, which led to lower cytotoxicity compared to pure ZnO NPs [79].

Due to the need to supply drugs to both hydrophilic and hydrophobic environments, it may be necessary to change the nature of the carrier surface. The increase of hydrophilic properties most often occurs by attaching carboxyl groups (-COOH). In this form, nanoparticles can be biofunctionalized to obtain modern nanocarriers. Using the addition of N-vinylpyrrolidone in the preparation of Ag NPs, it was possible to obtain a carrier for hydrophobic drugs in the aqueous medium, for example indomethacin—a non-steroidal anti-inflammatory drug with high drug loading efficiency of up to 95% [80]. Reznickova et al. compared the use of polyethylene glycol modified with various functional groups as a factor modifying AuNPs. The effect of applied modifications, i.e., nanoparticles with unmodified PEG, nanoparticles with polyethylene glycol (PEG) with attached thiol (PEG-SH) and amino (PEG-NH_2_) groups was investigated. Despite obtaining nanoparticles of similar size (1.5–5.9 nm) and of spherical shape, the presence of functional groups significantly influenced the toxicity of AuNPs. AuNPs stabilized with PEG-SH showed overall optimal physical and biological properties for drug delivery applications. They also showed no cytotoxicity in comparison with PEG and PEG-NH_2_ nanoparticles [81].

## 6. Summary

The rapid development of medicine, medical engineering, and other related fields has contributed to the development of new methods of pharmaceutical administration. The drug delivery system (DDS) includes the development of methods and tools that allow the administration of medicinal substances in a more effective and safe way.

It is postulated that the pharmaceutical carriers used meet a number of requirements regarding their physicochemical properties. They should be characterized by long-term action, as well as being constant over time and have a controlled release of the active substance. In addition, active substance carriers should be environmentally safe, biocompatible, biostable, and should not cause side effects. An important criterion is also the ease of production of drug carriers and their low price. These systems should not cause unwanted side reactions. A very important issue is the ability to deliver the active substance exactly to the destination and to maintain a constant concentration of the drug in the blood throughout the period of pharmacotherapy. Other requirements include convenient and easy application of these types of products. One of the most preferred methods is the use of metallic and oxide nanoparticles as carriers for transporting active substances.
